# Broadband terahertz absorber based on multi-band continuous plasmon resonances in geometrically gradient dielectric-loaded graphene plasmon structure

**DOI:** 10.1038/s41598-018-21705-2

**Published:** 2018-02-19

**Authors:** Jiawen Yang, Zhihong Zhu, Jianfa Zhang, Chucai Guo, Wei Xu, Ken Liu, Xiaodong Yuan, Shiqiao Qin

**Affiliations:** 1 College of Advanced Interdisciplinary Studies, National University of Defense Technology, Changsha, 410073 People’s Republic of China; 2 State Key Laboratory of High Performance Computing, National University of Defense Technology, Changsha, 410073 People’s Republic of China

## Abstract

We propose a broadband terahertz absorber consisting of nonstructured graphene loaded with arrays of elliptic dielectric cylinders. The relative bandwidth for the absorption above 90% reaches about 65%. The working mechanism of broad bandwidth mainly comes from two aspects. One is that the nonstructured graphene loaded with elliptic dielectric cylinders provides multiple discrete graphene plasmon resonances with large relative frequency interval. The other is that, for each discrete resonance, there exists a set of continuous plasmon resonances because the width of the dielectric structure varies continuously and gradiently. The broadband terahertz absorber we demonstrate here, based on geometrically gradient dielectric structures and nonstructured graphene, avoids the graphene processing, which shows great potential applications in related devices.

## Introduction

Terahertz (THz) devices have attracted increasing attention due to the unique properties of the THz region (0.1–10 THz), which share similarities with both the microwave and the far infrared ranges^1^. Many applications of THz devices have been proposed so far, such as communicating, spectroscopy, sensing and imaging^2–4^. Among these attempts, the absorber plays an important role in many devices working in THz range, including detectors^5^, sensors^6^, modulators^7^, thermal emitters^8^, camouflage devices^9^, etc. Traditionally, metallic metamaterials are the key components of many THz absorbers, structures of various geometries have been designed to realize different absorption functions such as dual-band^10,11^, multi-band^12,13^ and broadband^14–16^. Recently, graphene, a single-layered carbon atom arranged in a honeycomb lattice, has become one of the most promising materials for designing THz absorbers due to its tunability, broadband response and high carrier mobility^17^. Various types of graphene-based absorbers have been proposed to achieve near-unity absorption. Many of these absorbers are based on periodically structured graphene like disks^18^, microrings^19^, fishnets^20^, ribbons with gradient width^21^ and multilayered ribbons with asymmetric voids^22^. Nevertheless, edge effects of structured graphene are evident disadvantages of these structures^23^. To avoid this problem, absorbers using nonstructured graphene are put forward, such as metal-dielectric-graphene sandwich structure^24^, graphene loaded with periodical arrays of dielectric bricks^25^, multilayer graphene sheets on quartz substrates^26^, graphene with metasurface comprising of plasmonic structures^27^, and multilayer graphene with uneven dielectric slab structure^28^.

In this letter, we propose an alternative broadband mechanism of multi-band continuous plasmon resonances, and demonstrate that the relative bandwidth of over 90% absorption can reach about 65% in the THz range by using nonstructured graphene loaded with geometrically gradient dielectric structures. Here, we expound the physical mechanism firstly. Then, we present numerical simulations and results. Finally, we investigate the effects of some relative parameters.

## Structure and Physical Mechanism

**Figure 1:**
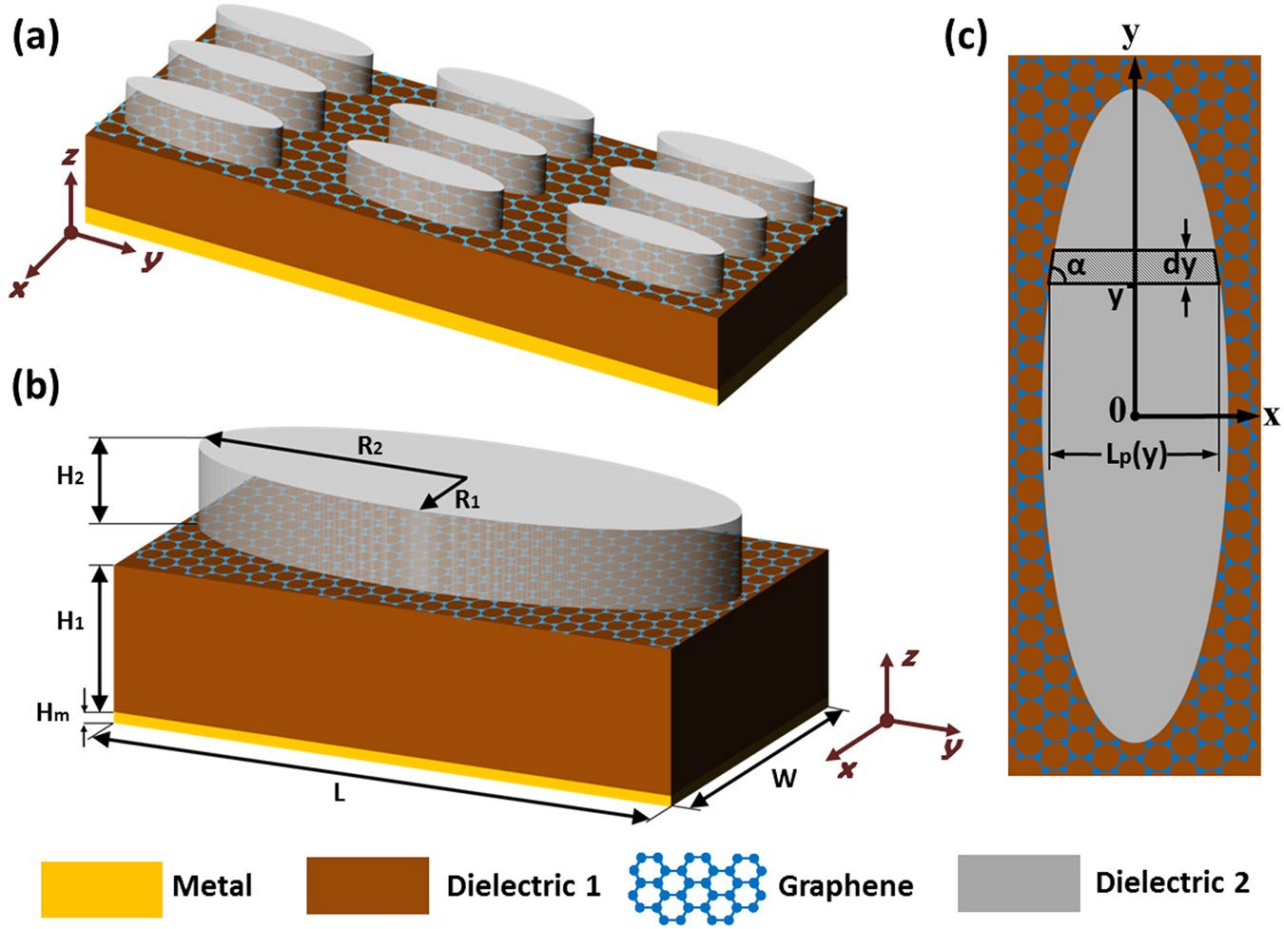
Broadband THz absorber using periodic arrays of elliptic dielectric cylinders loaded with graphene. (**a**) Schematic representation of the broadband THz absorber, consisting of periodic arrays of elliptic dielectric cylinders loaded with monolayer graphene supported by a piece of dielectric substrate on a thick piece of metal. (**b**) Schematic representation and geometrical characters of a unit cell. (**c**) Top view of a unit cell. The elliptic dielectric cylinders can be divided into infinite infinitesimals with the profile of isosceles trapezoids along the y-axis, the base and base angles of which are expressed by *L*_*p*_(*y*) and *α*, respectively. Each pair of lateral faces along the x direction of the infinitesimals form two reflective mirrors.

The proposed structure of the broadband THz absorber is presented in Fig. 1(a), which consists of periodic arrays of geometrically gradient dielectric loaded with monolayer graphene supported by a piece of dielectric substrate on a metallic film. In this design, the elliptic dielectric cylinders refer to the geometrically gradient dielectric. Figure 1(b) depicts geometrical parameters of a unit cell. The structure is characterized by the periodic interval *W* along x-axis, the periodic interval *L* along y-axis, the semi-minor axis *R*_1_, the semi-major axis *R*_2_ and the thickness *H*_2_ of the elliptic dielectric cylinder, the Fermi level *E*_*F*_ of graphene, the thickness *H*_1_ of the dielectric substrate and the thickness *H*_*m*_ of the metallic film. The material of the dielectric substrate and the elliptic dielectric cylinder are dielectric 1 and dielectric 2, respectively. In this structure, the metallic film can reflect waves and the nonstructured graphene loaded with periodic arrays of geometrically gradient dielectric can also provide array local resonant reflection (ALRR)^29^, which form two mirrors of an asymmetric Fabry-Perot (FP) cavity^27,30^. The transmission of waves can be blocked as long as the metallic mirror is thick enough compared with the typical skin depth at THz frequencies. The reflection of waves can be eliminated if the critical coupling condition is satisfied^27^ or the impedance of the absorber is matched to that of the free space^31^. When both the transmission and reflection channels are suppressed, the near-unity absorption can be realized. Since the near-unity absorption is decided by the critical coupling condition or impedance matching condition that is closely related with the graphene plasmon resonances (GPRs), we can broaden the absorption band through broadening GPRs. However, generally, the GPRs are narrowband. So, in order to achieve broadband absorption, the key problem is to find a method to broaden the GPRs. In this configuration, the GPRs are related to the formation of standing-wave patterns of graphene plasmons with the wave vector^32,33^.

1 $${k}_{GP}(\omega )=\frac{\pi {\hslash }^{2}}{{e}^{2}{E}_{F}}{\varepsilon }_{0}({\varepsilon }_{r1}+{\varepsilon }_{r2})\omega (\omega +{i\tau }^{-1})$$

where *e* is the charge of electron, *ħ* is the reduced Planck’s constant, ω is the angular frequency of graphene plasmons, *E*_*F*_ is the Fermi level of graphene, *τ* is the carrier relaxation time in graphene, *ε*_0_ is permittivity of vacuum, *ε*_01_ is the relative permittivity of the dielectric substrate, and *ε*_02_ is the relative permittivity of dielectric cylinders above the graphene film. When the structures are illuminated by a plane wave with the electric field linearly polarized along the x direction, the excited graphene plasmons propagate along the x direction and experience multiple reflections from the interfaces between the dielectric cylinders and air gap. In this structure, based on the thin element approximation in scalar Fourier optics, the elliptic dielectric cylinders can be divided into infinite infinitesimals with the profile of isosceles trapezoids along the y-axis, as shown in Figure 1(c). As a result, each pair of lateral faces along the x direction of infinitesimals act as two reflective mirrors of a FP cavity and FP-type resonances involving graphene plasmons occur. The distance *L*_*p*_(*y*), namely the FP cavity lengths, between two mirrors at a specific position y is approximated as

2 $${L}_{p}(y)=2{R}_{1}\sqrt{1-\frac{{y}^{2}}{{R}_{2}^{2}}}$$

The graphene plasmon resonance condition can be written as:

3 $$\mathrm{Re}({k}_{GP})\cdot 2{L}_{p}(y)=2\pi N+2\varphi $$

where Re(*k*_*GP*_) is the real part of wave vector *k*_*GP*_, *N* is a positive integer determining the order of a resonance mode, *ϕ* is the phase of the reflection coefficient for graphene plasmon reflection at two mirrors. In general, *ϕ* is non-zero that depends on the structural, material parameters and position *y*. The physical explanation for this non-zero f is related to extension of the plasmon field beyond the interfaces of the dielectric cylinders and air gap.

From eqs (1)–(3), by simple algebra operation, the resonant angular frequency for the N-th order plasmon resonance mode can be obtained as

4 $${\omega }_{N}=\frac{e}{\hslash }\sqrt{\frac{{E}_{F}(N\pi +\varphi ){R}_{2}}{2\pi {\varepsilon }_{0}{R}_{1}({\varepsilon }_{r1}+{\varepsilon }_{r2})\sqrt{{R}_{2}^{2}-{y}^{2}}}}$$

From equation (4), we find this configuration can provide multiple discrete GPRs since *N* can be set as different positive integers. We can also see that, for each discrete resonance, there exists a set of corresponding continuous plasmon resonances in the structure because *y* varies continuously in the elliptic dielectric cylinders. Under certain conditions, the frequency region of the continuous resonances can be larger than the corresponding frequency interval between two discrete neighbor resonances. So, the overlapping resonant frequencies can spread over a wide range, which is referred to as multi-band continuous GPRs.

As we know, the angle *β* between two mirrors of a FP cavity is a key factor determining the performance of resonance, and an angle of zero or close to zero is a condition for obtaining strong resonances. In our proposed structure, the angle *β* between two mirrors at a specific position *y* can be approximately given by

5 $$\beta (y)=\pi -2{\tan }^{-{\rm{1}}}(\frac{{R}_{2}}{{R}_{1}}\sqrt{{(\frac{{R}_{2}}{y})}^{2}-1})$$

From equation (5), we can find that, for a fixed *R*_1_ and *R*_2_, the angle *β* decreases with a decrease of position *y*, which shows the FP-type resonances are most likely to occur at smaller position *y* and can always occur at position *y* = 0 particularly. For a fixed *R*_1_ and position *y*, the angle *β* becomes smaller and tends to zero as *R*_2_ increases. This implies that it is reasonable to use the elliptic dielectric cylinders rather than usual dielectric cylinders to act as the geometrically gradient dielectric to obtain multi-band continuous GPRs. Since the near-unity absorption is closely related with the GPRs, the achieved multi-band continuous GPRs imply that the broadband near-perfect absorption may be obtained under certain conditions.

## Methods

To verify the theoretical prediction, we next conduct full-wave numerical simulations employing frequency domain solver in CST Microwave Studio. In the simulation, *ε*_*r*1_ = 4 (dielectric 1) and *ε*_*r*2_ = 12 (dielectric 2) are selected to show the physical mechanisms of broadband absorption. In the THz range, *ε*_*r*1_ = 4 and *ε*_*r*2_ = 12 can be approximatively provided by silicon dioxide and silicon, respectively^34^. The metallic material in this structure is gold, which is described by Drude model. The relative permittivity of gold is expressed by ${\varepsilon }_{gold}(\omega )={\omega }_{\infty }-$ ${\omega }_{P}^{2}/({\omega }^{2}+i\omega \gamma )$. Here, *ε*_∞_, *ω*_*P*_ and *γ* are 1.0, 1.38 × 10^16^ rad ⋅ *s*^−1^, and 1.23 × 10^13^ s^−1^, respectively^29^. Graphene is modeled as an anisotropic layer with thickness *h*_*g*_ = 1 nm. The relative permittivity of graphene comprises the out-of-plane component *ε*_*out*_ = 2.5 and the in-plane component *ε*_*in*_ = 2.5 + *iσ*(*ω*)/(*ωε*_0_*h*_*g*_), where the graphene optical conductivity *σ*(*ω*) is derived using the random-phase approximation (RPA)^35,36^. *σ*(*ω*) is calculated by *σ*(*ω*) = *σ*_*inter*_(*ω*) + *σ*_*intra*_(*ω*), where *σ*_*inter*_(*ω*) and *σ*_*intra*_(*ω*) are the interband and intraband contributions, respectively. In the THz range, the interband part *σ*_*inter*_(*ω*) is negligible compared with the intraband part *σ*_*intra*_(*ω*)^37^, hence we can use *σ*_*intra*_(*ω*) to approximate *σ*(*ω*) here, which is described as

6 $$\sigma (\omega )\approx \frac{2{k}_{B}{e}^{2}T}{\pi {\hslash }^{2}}\frac{i}{\omega +i{\tau }^{-1}}{\rm{In}}\mathrm{[2}\,\cosh ({E}_{F}\mathrm{/2}{k}_{B}T)]$$

where *ω* is the frequency of the incident wave, *k*_*B*_ is the Boltzmann constant, *e* is the charge of an electron, *T* = 300 K is the temperature, & planck; is the reduced Planck’s constant, *τ* is the carrier relaxation lifetime, and *E*_*F*_ is the Fermi level. Here, relaxation lifetime is expressed by $\tau =\mu {E}_{F}/e{v}_{F}^{2}$^27^, where the mobility *μ* is 10000 cm^2^/(V ⋅ s) and the Fermi velocity *v*_*F*_ is 10^6^ m/s. In the simulation, the open boundary condition is adopted in z direction and the unit cell boundary condition is adopted in x and y directions. The absorption is calculated from the obtained S parameters using A = 1 − R − T, where R is the reflection and T is the transmission.

## Results and Discussion

**Figure 2:**
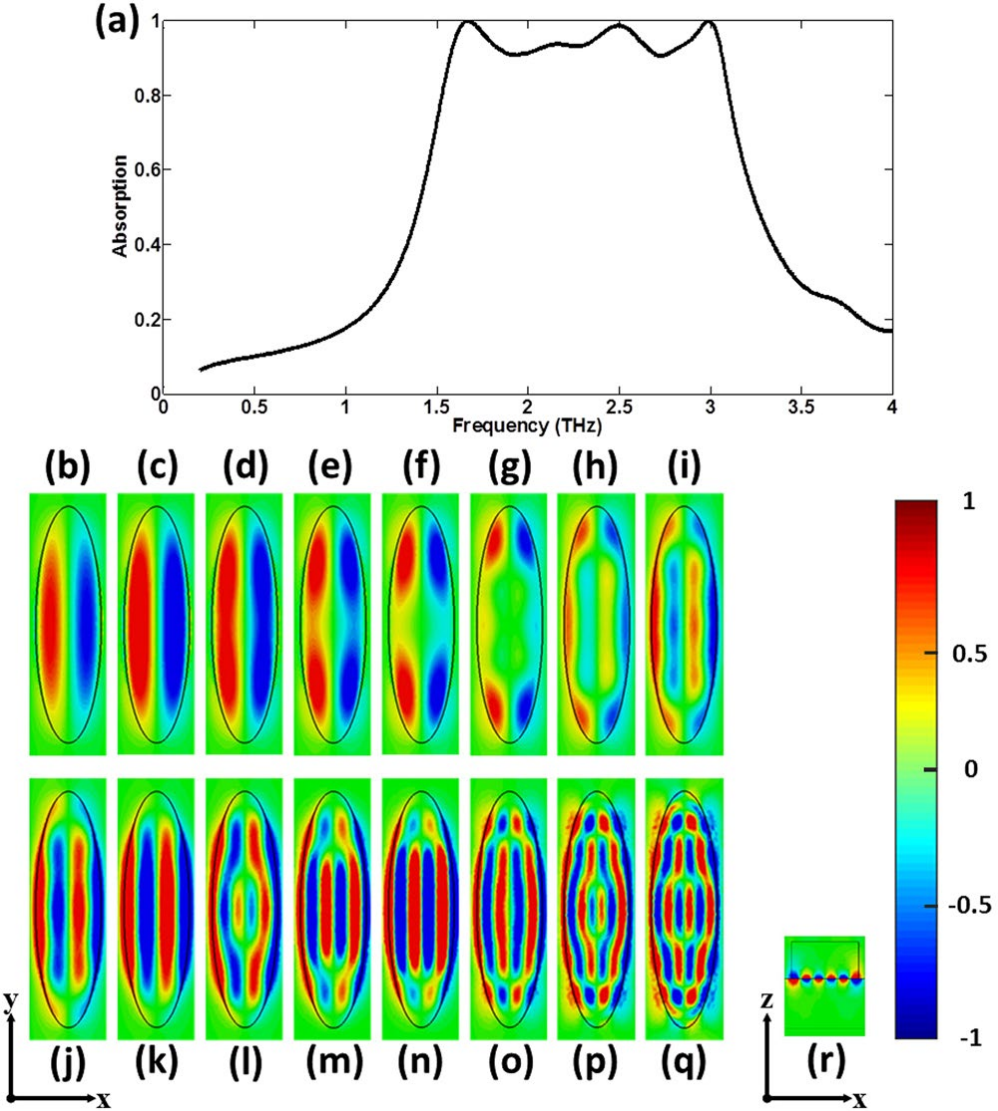
Broadband absorption based on multi-band continuous plasmon resonances. (**a**) The absorption spectrum under normal incident wave with the electric field parallel to x-axis. Typical distributions of the z component of the electric field at the distance 50 nm above the interface between the graphene and the elliptic dielectric cylinders at (**b**) 1.57 THz, (**c**) 1.67 THz, (**d**) 1.75 THz, (**e**) 1.80 THz, (**f**) 1.84 THz, (**g**) 1.95 THz, (**h**) 2.03 THz, (**i**) 2.08 THz, (**j**) 2.13 THz, (**k**) 2.32 THz, (**l**) 2.50 THz, (**m**) 2.60 THz, (**n**) 2.70 THz, (**o**) 2.89 THz, (**p**) 2.99 THz, (**q**) 3.07 THz, respectively. (**r**) Distribution of the z component of the electric field on the central cutting x-z plane at 3.07 THz.

First, we consider the case where *E*_*F*_ = 0.5 eV, *H*_1_ = 15 *μ*m, *H*_2_ = 11 *μ*m, *H*_*m*_ = 2 *μ*m, *R*_1_ = 10 *μ*m, *R*_2_ = 36 *μ*m, *L* = 80 *μ*m, *W* = 24 *μ*m. Figure 2(a) shows the corresponding absorption spectrum under normal incident wave with the electric field parallel to x-axis. From Fig. 2(a), we can see the above 90% absorption covers the frequency range of 1.57–3.07 THz and the relative bandwidth reaches about 65%. In order to intuitively confirm the broadband mechanism, the typical distributions of the z component of the electric field at the distance 50 nm above the interface between the graphene and the elliptic dielectric cylinders for sixteen sample frequencies in the 90% absorption band are calculated and shown in Fig. 2(b)–(q), respectively. Fig. 2(b)–(q) clearly show that the different absorption frequencies correspond to the different electric field patterns. However, we can find that, although these field patterns are different, according to the number of nodes in x direction they can be divided into several categories. For example, all the field patterns in the frequency range of 1.57–1.95 THz (Fig. 2(b)–(g)) are similar and have one node in x direction, which shows all the corresponding modes are the first-order graphene plasmon resonance, namely the dipole resonances. So, we describe the frequency range of 1.57–1.95 THz as the first-order continuous absorption band. In the absorption band, the different absorption frequencies correspond to the different positions along y direction in elliptic dielectric cylinder, which possess the different FP cavity lengths. Similarly, the high order continuous absorption band can be formed, as shown in Fig. 2(h)–(q). It is noteworthy that the field patterns at some frequencies are not easy to distinguish because of the overlapping of different absorption bands. For example, at the frequency of 3.07 THz, we can see that the field patterns contain three GPRs occurring at the different positions, which are the second, third and fourth order mode, respectively. Figure 2(r) shows the distribution of the z component of the electric field on the central cutting x-z plane at 3.07 THz. From Fig. 2(r), we can see the field is bound to the interface between the dielectric and graphene, which further verifies the absorption is closely related with the GPRs. These results show that our simulated results and theoretical predictions are in good agreement, which confirms the broadband mechanism.

In order to investigate the characteristics of the broadband THz absorber, we sweep a few parameters, including semi-major axis *R*_2_, incident angle *θ* and Fermi level *E*_*F*_. It is necessary to stress that in each group of simulations, all the other parameters and conditions are kept the same as the above simulations.

**Figure 3:**
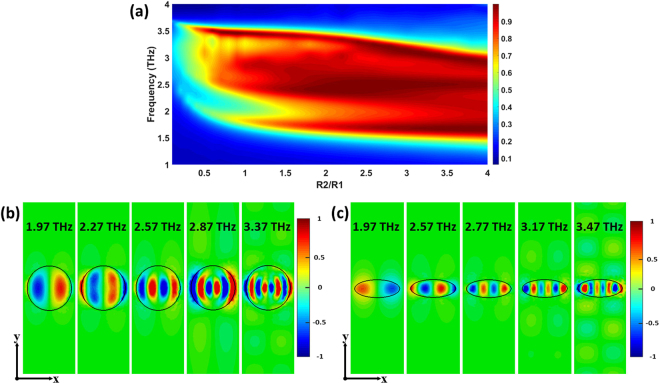
The effect of the ellipticity of elliptic dielectric cylinders on the absorption. (**a**) Calculated absorption as a function of frequency and ellipticity *R*_2_/*R*_1_ under normal incident wave with the electric field parallel to x-axis. (**b**) Typical distributions of the z component of the electric field at the distance 50 nm above the interface between the graphene and the elliptic dielectric cylinders for *R*_2_/*R*_1_ = 1 at 1.97, 2.27, 2.57, 2.87 and 3.37 THz, respectively. (**c**) Typical distributions of the z component of the electric field at the distance 50 nm above the interface between the graphene and the elliptic dielectric cylinders for *R*_2_/*R*_1_ = 0.4 at 1.97, 2.57, 2.77, 3.17 and 3.47 THz, respectively.

We first investigate the effect of ellipticity of elliptic dielectric cylinders by varying *R*_2_ from 1 to 40 *μ*m while fixing *R*_1_ = 10 *μ*m. Figure 3(a) shows the corresponding absorption as a function of frequency and *R*_2_/*R*_1_ under normal incident wave with the electric field parallel to x-axis. It can be observed that the absorption is very sensitive to ellipticity *R*_2_/*R*_1_. When the values of *R*_2_/*R*_1_ are very large (e.g. *R*_2_/*R*_1_ > 3), the characteristics of broadband absorption maintain well. On the other hand, when *R*_2_/*R*_1_ is relatively small (e.g. *R*_2_/*R*_1_ < 1.5), the broadband absorption tends to be torn. This is in agreement with that, decreasing the ellipticity *R*_2_/*R*_1_ results in an increase of the angle *β* between two mirrors of FP-type GPRs at specific positions in the elliptic dielectric cylinders, which eventually makes the corresponding resonance strength weaker and even disappearing. As a result, the corresponding absorption becomes small and the broadband absorption is torn. In order to further confirm the broadband mechanism, the typical distributions of the z component of the electric field at the distance 50 nm above the interface between the graphene and the elliptic dielectric cylinders for *R*_2_/*R*_1_ = 1 and *R*_2_/*R*_1_ = 0.4 are shown in Fig. 3(b) and (c), respectively, which are significantly different from Fig. 2(b)–(q) where *R*_2_/*R*_1_ = 3.6. Comparing the typical distributions of electric field in three different *R*_2_/*R*_1_ ratios, we can visually find that the larger *R*_2_/*R*_1_ can really provide more GPRs. For example, at the case of *R*_2_/*R*_1_ = 0.4, the FP-type GPRs only occur at the position *y* = 0. However, for *R*_2_/*R*_1_ = 3.6, the FP-type GPRs can occur at a wide region of position *y*. These field distributions are consistent with the prediction.

**Figure 4:**
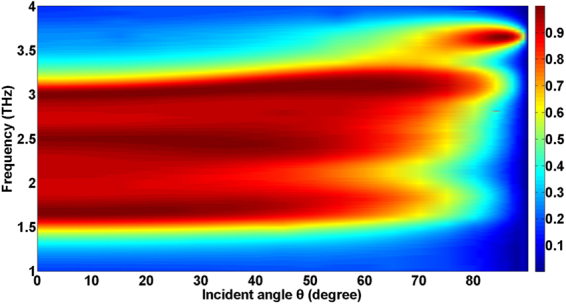
The effect of the incident angle on the absorption. Calculated absorption as a function of frequency and incident angle *θ* while maintaining the incident plane wave in the x-z plane and the magnetic field parallel to y-axis.

Next, we vary the incident angle *θ* from 0° to 89.9° while maintaining the incident plane wave in the x-z plane and the magnetic field parallel to y-axis to examine the incident angular sensitivity. The incident angle *θ* is defined as the angle between the incident plane wave and the positive z-direction. Figure 4 shows the calculated absorption as a function of frequency and *θ*. It can be seen that the dependence of the absorption on the incident angle is relatively weak when the incident angle varies between 0° and 60°. The reason for this is that the near-unity absorption of this type of absorber is closely related to the GPRs. However, the excitation of the GPRs is insensitive to the incident angle^38^. As the incident angle increases beyond 60°, the absorption decreases rapidly. This can be understood through the following qualitative analysis. The near-unity absorption is also closely related to destructive interference. But, in the case of large angle incidence, the reflection amplitudes from two mirrors of the asymmetric FP cavity formed by the metallic film and the nonstructured graphene loaded with periodic arrays of geometrically gradient dielectric have a large mismatch, and the destructive interference amplitude-matching condition is destroyed. It is noticeable that there is an abnormal absorption phenomenon near the incident angle 85°. The reason of this phenomenon is: for the structure consisting of a single layer of lossy two-dimensional (2D) material separated from a mirror by a dielectric spacer layer, there always exists an angle of incidence, at which the external radiation decay equals to internal absorption decay and thus complete absorption of the 2D material can be achieved^39^. For our absorber, at 3.6 THz, the above condition of complete absorption is satisfied when the incident angle is near 85°.

**Figure 5:**
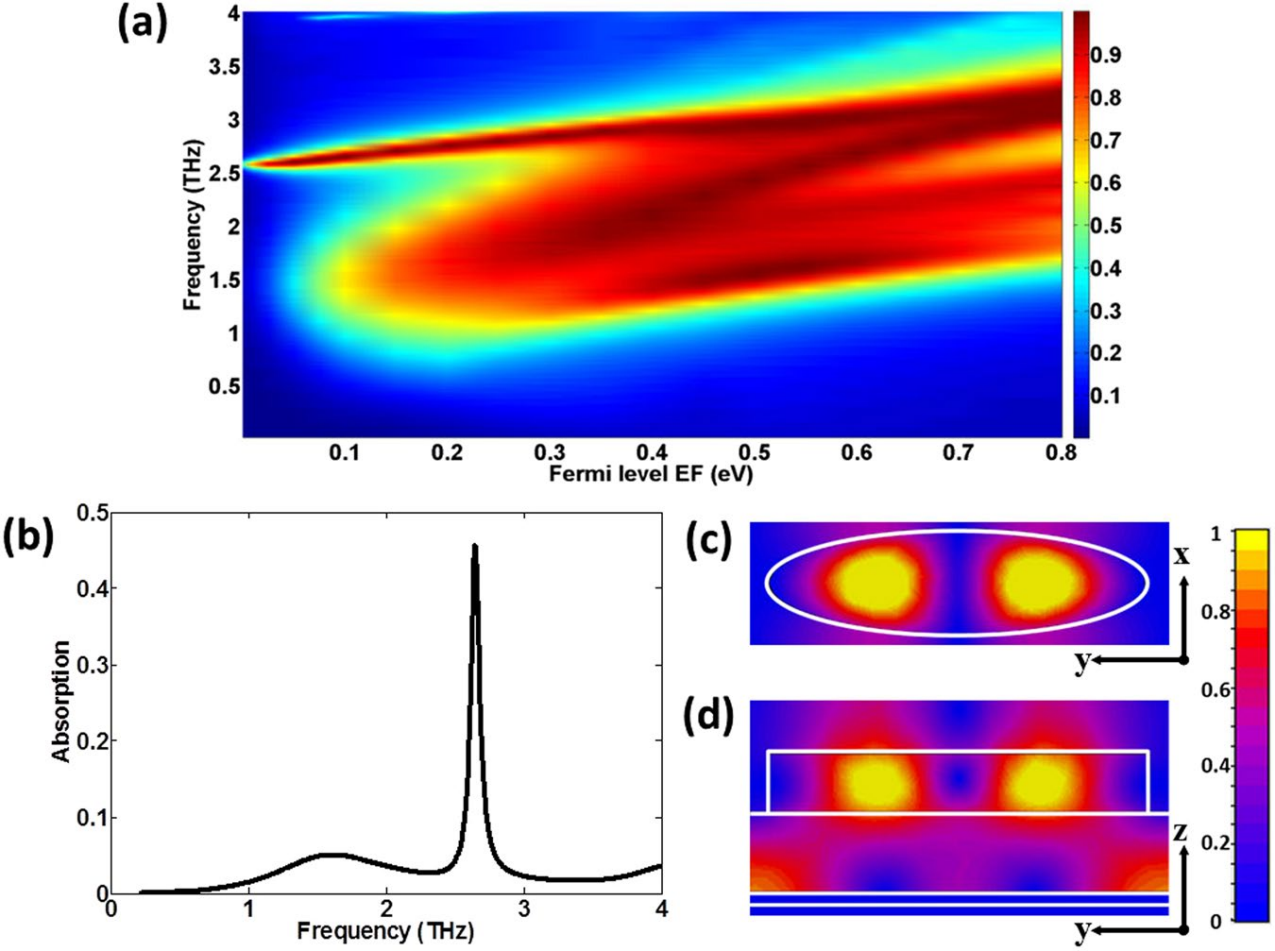
The effect of the Fermi level of graphene on the absorption. (**a**) Calculated absorption as a function of frequency and Fermi level *E*_*F*_. (**b**) The absorption spectrum for *E*_*F*_ = 0.0 eV under normal incident wave with the electric field parallel to x-axis. (**c**) Magnetic field amplitude patterns on the central cutting x-y plane of the dielectric cylinder for *E*_*F*_ = 0.0 eV at 2.64 THz. (**d**) Magnetic field amplitude patterns on the central cutting y-z plane for *E*_*F*_ = 0.0 eV at 2.64 THz.

Then, we show the effect of Fermi level *E*_*F*_ on the absorption in Fig. 5(a). From Fig. 5(a), we can see that there is a blueshift of the absorption band as *E*_*F*_ increases, which is because the plasmon resonance frequencies of the doped graphene increase with increased Fermi level^40^. It can also be seen that the absorption decreases rapidly when *E*_*F*_ varies from 0.2 to 0 eV, which results from the fact that the metallic character of graphene decreases when *E*_*F*_ is relatively small. However, there is an interesting and abnormal phenomenon that a narrowband absorption still exists near the upper absorption band even as *E*_*F*_ approaches 0, where GPRs do not exist. To explain this phenomenon, we extract the absorption for *E*_*F*_ = 0.0 eV from Fig. 5(a), as shown in Fig. 5(b). From Fig. 5(b), we can find that the absorption peak frequency equals to about 2.64 THz. The magnetic field amplitude distributions for 2.64 THz are presented in Fig. 5(c) (central cutting x-y plane of the dielectric cylinder) and Fig. 5(d) (central cutting y-z plane). We can clearly see the field is mainly concentrated within the dielectric cylinder, which is very different from the field of GPRs bound to the interface between the dielectric and graphene (see Fig. 2(r)). This shows that the abnormal absorption is attributed to the dielectric resonance along the y-axis in elliptic dielectric cylinders.

## Conclusions

In summary, we propose a broadband THz absorber based on multi-band continuous plasmon resonances sustained by geometrically gradient dielectric loaded with graphene. The simulation results show that the relative bandwidth for the absorption above 90% can reach about 65%. The broad bandwidth mainly originates from two key aspects. One is that the nonstructured graphene loaded with geometrically gradient dielectric can provide multiple discrete graphene plasmon resonances with large relative frequency interval. The other is that, for each discrete resonance, there exists a set of continuous plasmon resonances because the width of dielectric structure varies continuously and gradiently. Besides, FP resonances of the dielectrics also contribute to the absorption. Based on nonstructured graphene, the absorber avoids processing graphene and destroying the unique properties of graphene. The approach of combining geometrically gradient dielectric structures and nonstructured doped graphene facilitates the application of the broadband THz absorbers and related devices.
